# Enhancement of early warning properties in the Kuramoto model and in an atrial fibrillation model due to an external perturbation of the system

**DOI:** 10.1371/journal.pone.0181953

**Published:** 2017-07-28

**Authors:** David García-Gudiño, Emmanuel Landa, Joel Mendoza-Temis, Alondra Albarado-Ibañez, Juan C. Toledo-Roy, Irving O. Morales, Alejandro Frank

**Affiliations:** 1 Centro de Ciencias de la Complejidad, Universidad Nacional Autónoma de México, México D.F., México; 2 Instituto de Ciencias Nucleares, Universidad Nacional Autónoma de México, México D.F., México; 3 Laboratorio Nacional de Ciencias de la Complejidad, México D.F., México; 4 Instituto de Fisiología Celular, Benemérita Universidad Autónoma de Puebla, Puebla, México; Indiana University, UNITED STATES

## Abstract

When a complex dynamical system is externally disturbed, the statistical moments of signals associated to it can be affected in ways that depend on the nature and amplitude of the perturbation. In systems that exhibit phase transitions, the statistical moments can be used as Early Warnings (EW) of the transition. A natural question is thus to wonder what effect external disturbances have on the EWs of system. In this work we study the impact of external noise added to the system on the EWs, with particular focus on understanding the importance of the amplitude and complexity of the noise. We do this by analyzing the EWs of two computational models related to biology: the Kuramoto model, which is a paradigm of synchronization for biological systems, and a cellular automaton model of cardiac dynamics which has been used as a model for atrial fibrillation. For each model we first characterize the EWs. Then, we introduce external noise of varying intensity and nature to observe what effect this has on the EWs. In both cases we find that the introduction of noise amplified the EWs, with more complex noise having a greater effect. This both offers a way to improve the chance of detection of EWs in real systems and suggests that natural variability in the real world does not have a detrimental effect on EWs, but the opposite.

## 1 Introduction

One of the most important properties that is common to complex systems is the presence of critical thresholds in its dynamics [1] at which the system shifts abruptly (relative to its own long-range time scales) from one state to another. These abrupt changes in the state of a system occur near bifurcation points. At a bifurcation, the state of the system becomes unstable and the system jumps to an alternative (usually radically different) stable state. Stable states are related to a minimum in the potential energy and are separated by a potential barrier associated with unstable states. The point at which the bifurcation begins is called a critical point (CP). At this point the potential barrier flattens almost to the point of disappearing, giving place to a phenomenon known as critical slowing down (CSD) [1], the fact that the recovery time of a system after a perturbation increases when the system approaches a bifurcation in the dynamics. There are several examples of this across many fields: in medicine, asthma attacks or epileptic seizures; in global finances, in the vicinity of market crashes; in Earth systems, abrupt shifts in climate may occur or catastrophic changes in ecosystems.

There has been recently a growing interest in understanding how a complex system behaves in the vicinity of catastrophic shifts, in part to predict and possibly to control the timing and evolution of such transitions [2–6]. The search for indicators that can predict these shifts has been quite fruitful, in particular with the discovery of the so-called early warning (EW) signals [1], which are related to the CSD phenomenon. One of the most useful procedures is to study the time evolution of a representative variable of the system; this is known as a time series.

When the time series is modeled as a stochastic process it is possible to estimate the underlying probability distribution that originates it and, in principle, if the system approaches a critical point (CP) due to CSD phenomenon several moments of this probability distribution will change, providing a way to understand the dynamics of the system [2–5]. For instance, the second moment of the distribution, the variance, will diverge because a system will recover very slowly from perturbations when it is close to a critical threshold, which in principle allows the system to drift across different states [3, 7]. Depending on the system, the fluctuations could be asymmetric if the system is near configurations with an unstable equilibrium. This will produce changes in the third moment of the distribution, the skewness [4, 7]. Changes in the fourth moment of the distribution are also possible since near criticality the system will visit extreme states more often [5, 7].

As noted in [8] it is necessary to cross check if the autocorrelation of the time series is also affected. An analysis of the power spectral density (PSD) is also useful because when a system is in a CP all scales become important for the system dynamics. This is reflected in the fact that the system becomes scale invariant, and the PSD takes the shape of a power law [7].

The combination of all these tools can help detect whether the system is about to undergo a critical transition, but sometimes the behavior of the EWs in the vicinity of a critical point is not clear enough to predict the transition with enough foresight. As is shown in [9–11] (for the specific case of the Kuramoto model) when a perturbation is introduced to the system the variance of the time series is affected in a way that depends on the nature and the amplitude of the perturbation. This leads naturally to wonder whether the perturbation will also affect the behavior of the EWs, and if so, how the effect depends on the nature and the amplitude of the perturbation. Additionally, would the introduction of a perturbation to the system improve our ability to identify EWs? Because of these unknowns it is important to better understand the effects of perturbations on the EWs, as improved detection could lead to earlier or clearer indication of the impending transition. The purpose of this work is to determine the effects of external perturbations on the EWs, studying the importance of their amplitude and complexity, and to determine what kind of EWs are more sensitive to this effect.

To answer these questions we have explored two models for which Early Warnings are known to exist:

1. The Kuramoto Model [11, 12], which provides a simple theoretical framework to study how synchronization may emerge spontaneously in the dynamics of a many-body interacting system. This model is widely used in biology to study the behavior of very different systems, including fireflies and even neurons.
2. A cellular automaton model of electrical conduction in cardiac atrial tissue, which is capable of simulating atrial fibrillation caused by reentrant circuits [13]. The simplicity of this model has allowed its authors to suggest methods of diagnosis and treatment for this disease.

The work is organized as follows: in Section 2 we briefly describe the Kuramoto Model, followed by a description of the atrial fibrillation model in Section 3. In Section 4 we present our analysis of the EWs and discuss the effects of adding a perturbation to these systems. Finally, in Section 5 we conclude the present work.

## 2 The Kuramoto model

The Kuramoto model is a paradigm in biological systems dealing with synchronization, such as colonies of fireflies switching on and off and the firing pattern of brain cells during a cognitive process. It consists of a population of *N* coupled oscillators characterized by their individual time-varying phases *θ*_*i*_(*t*) and natural frequencies *ω*_*i*_, initially distributed with a given probability density *g*(*ω*) and with dynamics governed by

$${\dot{\theta }}_{i}=\omega_{i}+\overset{N}{\underset{j=1}{\sum }}K_{ij}\text{sin}\left(\theta_{j}-\theta_{i}\right),\;i=1,\ldots ,N,$$ (1)

where *K*_*ij*_ represents the coupling constant between oscillators *i* and *j*.

Each oscillator tries to run independently at its natural frequency, while the coupling tends to synchronize it to all the others. When the coupling is weak enough, the oscillators run incoherently, while when it is above a certain threshold collective synchronization emerges. It is common to define two order parameters r and *ψ*, given by

$$\begin{array}{c} re^{i\psi }=\frac{1}{N}\sum_{j=1}^{N}e^{i\theta_{j}}. \end{array}$$ (2)

The parameter *r* is a measure of overall synchronization between the oscillators, and thus of the amount of collective behavior in the system, and the global phase *ψ* is connected to the average phase of all the oscillators. Under the assumption of a mean field, where it is assumed that all the oscillators have the same coupling constant *K*, Eq (1) can be rewritten as

$$\begin{array}{c} {\dot{\theta }}_{i}=\omega_{i}+Kr\sin(\psi -\theta_{i}),\;\;\;\;i=1,\ldots ,N. \end{array}$$ (3)

A perturbation can then be added to the system in the following way:

$$\begin{array}{c} {\dot{\theta }}_{i}=\omega_{i}+\xi_{i}+Kr\sin(\psi -\theta_{i}),\;\;\;\;i=1,\ldots ,N, \end{array}$$ (4)

where the perturbation term *ξ*_*i*_ is defined so that 〈*ξ*_*i*_(*t*)〉 = 0. This condition means that the time average of the perturbation acting on oscillator *i* is zero. In our case we have used a Gaussian distribution centered at zero, with the value of the standard deviation *σ* controlling the amplitude of the perturbation. For a more complete review of this model, a detailed explanation can be found in [11, 12].

As noted in [11, 12] this system suffers a critical transition between the totally incoherent state (*r* ≃ 0) and the synchronized one (*r* ≃ 1) at a specific value of the global coupling constant *K*. In this work we make use of the numerical solution of Eq 4 and analyze the resulting time series in order to study the presence of Early Warnings in the system. We simulated a system of 100 thousand oscillators for a range of the coupling constant *K* from 0 to 3.5, and explored the effect of different values of the amplitude of the noise term.

## 3 The atrial fibrillation model

The second model we have studied in this work is a model of electrical conduction in atrial tissue, which is described in detail in [13]. It is based on a cellular automaton and reproduces multiple well-known observed features of reentry fibrillation caused by connexins disconnection (In the original article this is related to fibrosis, but here we are simply interpreting it as the failure of electrical conduction between cells). The state conducive to fibrillation is established through an abrupt transition, giving us the opportunity to measure EWs near this change of dynamics.

This model is a two dimensional representation of a portion of atrial tissue of 20 cm^2^ with vertical cyclic boundary conditions which gives a cylindrical topology to the system. This is modeled computationally as a 200 × 200 grid of blocks, in which each block represents a set of five heart cells. Every block is connected horizontally to the ones to its sides in both directions (except for the first and the last column, which are only connected in one direction, respectively). The blocks are also connected vertically with probability *ν*, so in this case *ν* acts as the control parameter. For *ν* = 1, all the blocks are connected vertically which gives homogeneity to the tissue; however, as this parameter becomes smaller the tissue loses connectivity and this can cause (under certain circumstances described below) the elliptical wave fronts characteristic of AF [14].

**Fig 1:**
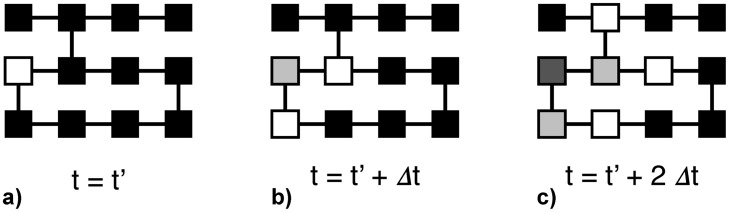
Example of the propagation rules of the cellular automaton to model atrial tissue. (a) At *t* = *t*′, an excited block (white) is surrounded by blocks at rest (black). At the next timestep *t* = *t*′ + Δ*t*, the blocks connected to the white block become excited and turn white, while the excited block enters into the refractory state (shown as progressively darker shades of grey) in which it can not be excited. At the following timestep *t* = *t*′ + 2Δ*t* the process is repeated, thus propagating the excitation forward. The duration of the refractory state is a parameter of the model and lasts multiple timesteps.

To simulate the propagation of an electrical pulse through the tissue we consider that the blocks can be in one of three states: at rest (black), excited (white) and in a refractory state (grey scale). At the beginning all the blocks are at rest. An excitation pulse is then introduced by exciting all blocks on the left boundary, simulating the action of the cardiac pacemaker. The pulse then propagates according to the following rules: 1) a block at rest that is directly adjacent (either vertically or horizontally) to an excited block becomes excited at the next timestep; 2) after a block has been excited, it enters a refractory period of predefined length in which it cannot be excited again; 3) once the refractory period ends, the block can be excited again. An example of these propagation rules is shown schematically in Fig 1. Due to its simplicity the model is only meant to simulate the propagation of an electrical pulse through the tissue; it does not capture the complexity of the action potentials that generate actual pulses in a real heart.

**Fig 2:**
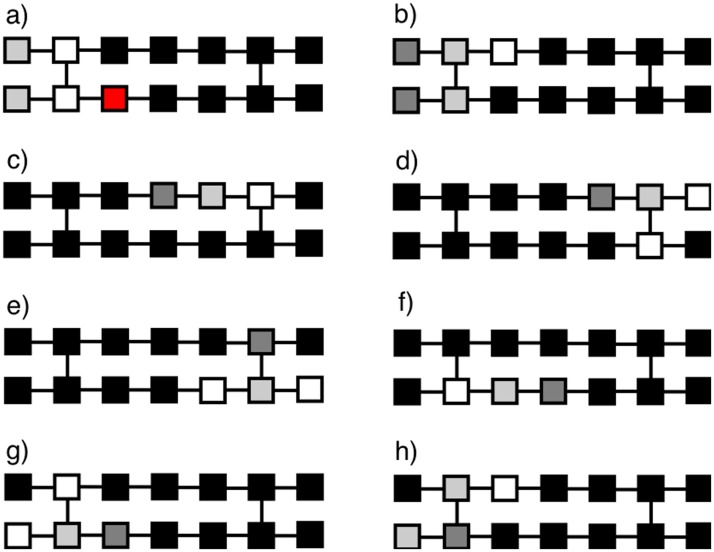
(a) An incoming pulse traveling from left to right encounters a block that fails to excite (shown in red). (b) The pulse continues normally in the upper row, but is blocked in the lower one. (c) The pulse reaches the vertical connection, which allows the pulse to travel back into the lower row (d), thus propagating backwards as shown in (e). Finally, when the backwards-traveling pulse reaches the first vertical connections, the process is repeated and this forms an elliptical pattern as shown in (f)–(h).

Aside from the parameter *ν*, two important factors are necessary to generate the self-sustaining elliptic wave fronts that are associated to atrial fibrillation. One is the fact that some blocks are made to stochastically fail to become excited, thus blocking the propagation of the pulse; this is interpreted as a failure of electrical conduction in the tissue. In the model, a fraction *δ* = 5% of cells are initially selected as being “defective”, having a probability *ϵ* = 5% of failing to become excited when they should. The second factor is the length of the refractory period. The elliptical wave fronts are generated when a block fails to excite and a vertical connection further down the direction of propagation permits the pulse to travel backwards in the parallel row. Then, if that backwards-traveling pulse encounters another vertical connection, and the refractory period of the block across the connection has had time to expire, a self-sustaining circuit of excitation is formed, giving rise to the elliptical wave fronts that are interpreted in the model as atrial fibrillation. It is worth mentioning that this mechanism for the generation of fibrillation does not depend on the rate at which pulses are imposed at the left boundary, being entirely due to the topology of the connections in the tissue and to the possibility of failure of electrical conduction (at least for the time scales involved in these simulations). In the model, all it takes for the continued generation of these waves is that the blocks in the circuit are at rest before the returning excitation wave reaches them again. In fact, some AF treatments work by increasing the refractory time in order to prevent the formation of the wave fronts [15]. The combination of the factors leading to the formation of elliptical wave fronts is shown in Fig 2.

In our study we performed 1000 runs for each fixed value of the parameter *ν* using a different random set of damaged cells and different random vertical connections for each run. We then varied *ν* from 0.02 to 0.25 in steps of 0.01 (the behavior above *ν* = 0.25 is qualitatively similar).

The last important parameter of the model is the rate at which pulses are introduced at the left boundary of the grid. In order to model external perturbations we have allowed the rate of these pulses to vary stochastically according to distributions that are closer to those encountered in real systems. Physiologically this could be understood as an extrasystole in the atrial node. We experimented with several types of rates:

1. To serve as a basis for comparison, we first followed the idea of the original article by generating the pulses at fixed intervals of 13.2 ms (as would an artificial pacemaker).
2. Then, in order to introduce external perturbations to obtain a more realistic simulation, we introduced stochastic variations in the pulse-to-pulse intervals. First, we obtained the intervals at random from a uniform distribution in the range 13.2 ± 1.3 ms.
3. Next the interval was chosen at random from a Gaussian distribution centered at 13.2 ms with a standard deviation of 1.3 ms.
4. Finally, we used real values of RR intervals taken from the Fantasia database (Note: The RR interval is defined as the time elapsed between two consecutive R waves in the electrocardiogram) [16]. This was done in the spirit of introducing the level of complexity found in actual biological systems, but not with the intention to replicate actual atrial function.

To obtain a measurable univariate signal from the simulations that bears some similarity to biological signals we proceed as follows. A fixed region in the middel of the grid of 200 x 50 blocks is chosen at the start. We assigned an arbitrary intensity to each gray scale of the pulse and then, at each iteration of the simulation the average intensity of the blocks in the region is computed and recorded. As a result a timeseries of the average intensity over the region is obtained. This signal can be interpreted as analogous to that obtained from the lead II configuration of an electrocardiogram (ECG), which is known to be a good measure of atrial electrical activity. The results presented in this study are obtained from the analysis of these time series. It should also be noted that RR intervals are used only to introduce variations in the time between pulses that exhibit complex statistics, as an attempt to approach the variability observed in biological systems; we only model atrial electrical activity in the study.

## 4 Results and discussion

### 4.1 Early warnings for the Kuramoto model

#### 4.1.1 Statistics-based early warnings

**Fig 3:**
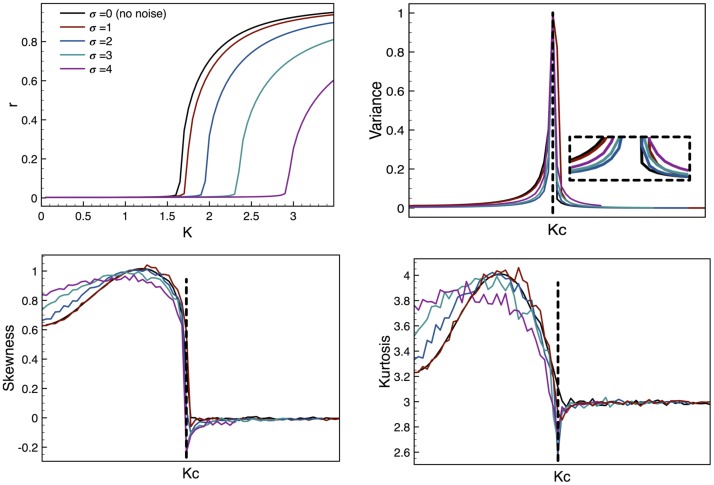
Statistical moments for the Kuramoto model. (a) Plot of the synchronization parameter *r* as a function of the coupling strength *K*. The effect of the perturbation is to shift the critical value of *K*, with an increasing effect for higher perturbation levels. (b) The variance is shown to diverge when approaching the transition. When approaching from higher values of *K* the effect of the perturbation is to soften the curve, enhancing the EW. The inset shows a detailed view of the beginning of the divergence, where we can see that for *K* > *K*_*C*_ the slope of the curve is smoother as the perturbation increases. (c) The skewness shows a dramatic change before and after the transition; for values of *K* before the CP its behavior is soft enough to consider it a good EW, and the same can be said for the kurtosis shown in (d).

Considering that the time series obtained through the model are modified stochastically by the introduction of the noise term (the external perturbation), we performed the EW analysis on the statistical moments over an ensemble for several values of the *σ* parameter, thus controlling the amplitude of the perturbation; the results are shown in Fig 3. In Fig 3a we show the parameter *r* as a function of the coupling constant *K*. This parameter is a measure of the fraction of oscillators that are synchronized, as discussed previously. It can be seen that the effect of the perturbation is to shift the behavior of synchronization to a higher value of *K*. This is to be expected, as it is more difficult to achieve a state of synchronization when there is an external stimulus that opposes it directly. Eventually, however, this state is reached and then the system behaves as if there were no perturbations at all. This result agrees with results obtained in [9, 10].

It is well known theoretically that the variance of a timeseries diverges when the system approaches a CP. Our simulations reproduce this result well (see Fig 3b), although the variance only reaches a finite maximum because our simulations have a finite number of oscillators. Since the perturbation has the same shifting effect on the position of the CP, we shifted the plots so that the CP (given by the value of *K*_*C*_) coincides for all values of *σ*. This offers a clear comparison of the effects of different intensities of perturbation both before and after the critical transition. As can be seen in Fig 3b, in every case the variance curve has the expected shape, increasing abruptly at the transition. Interpreting the behavior of this moment as an EW, we find that the effect of the perturbation is to slightly soften the decay of the variance after the transition (i.e. for *K* > *K*_*C*_), with the effect being more noticeable the larger the amplitude of the perturbation. Thus, the introduction of the perturbation makes this moment a more robust EW (at least on that side of the CP).

The third and fourth moments (skewness and kurtosis, respectively), reveal an interesting behavior (see Fig 3c and 3d): for values of *K* larger than the critical value the distribution approaches a Gaussian. This could be because in the synchronized state almost all the oscillators move as one and the few that are out of tune can be considered to follow this distribution. These two moments are useful as EWs in the sense that they have a different behavior before and after the transition. The change when approaching the CP from larger *K* is too drastic, so it is not quite an EW; but when the CP is approached from smaller *K*, these moments exhibit a soft transition, so one can interpret them to be good EWs.

#### 4.1.2 Memory based early warnings

**Fig 4:**
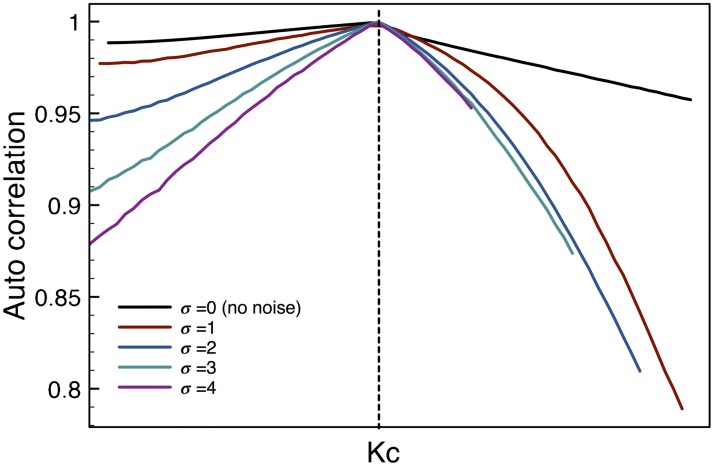
Lag-1 autocorrelation. At the CP the lag-1 autocorrelation approaches 1, indicating that the system has strong short-term memory. Moving away from the CP the system loses memory gradually but with different rates for each value of the perturbation amplitude *σ*, making of it a very good EW. For comparison purposes these plots were shifted so that the CP of each curve coincides, as explained before.

Another way to establish early warnings is study how the memory of the system changes during the transition. To do this we have analyzed the lag-1 autocorrelation, which is a measure of the short-term memory of the system. It is known that near a critical point the lag-1 autocorrelation approaches 1, which is an indication of high short-term correlation in the system. Fig 4 shows precisely this expected behavior as the CP is reached.

The effect of the perturbation is apparent in the behavior both before and after the CP. It can be seen that with increasing intensity of the perturbation the value of the lag-1 autocorrelation decreases more notably. Thus, the introduction of the perturbation also enhances this EW. This is an important result because even though the statistical moments are also improved as EWs the improvement is much stronger for the lag-1 autocorrelation. Furthermore, the effect is asymmetric with respect to the direction of approach to the CP, thus providing a way to tell which side of the CP and how far the system is from the transition.

**Fig 5:**
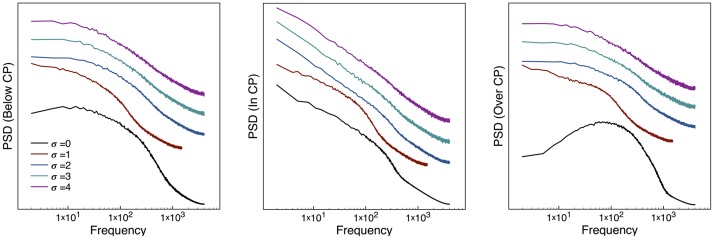
PSD of the Kuramoto model. The power spectrum is shown below (left), at (center) or above (right) the critical point, for different values of the perturbation amplitude. For the case *σ* = 0 the spectrum at the critical point is curved and can not be fitted cleanly by a power law. By adding a perturbation this curvature diminished, and the critical spectrum becomes much closer to a power law.

To study the long-range memory of the time series it is common to use the power spectral density (PSD). Near critical points the fluctuations of a system become scale invariant, and this is shown in the PSD as a power law [7]. Our results for this analysis are shown in Fig 5. First, in the case without a perturbation (*σ* = 0, lower black line) it can be seen that before the CP the spectrum is curved and can’t be fitted by a power law. As the system approaches the CP this curvature becomes less noticeable, approaching but not quite reaching a straight line. This is an indication that the system’s long-range memory is enhanced. After the CP is passed the curvature reappears, showing the loss of this memory. This behavior is also seen in the cases with a perturbation, but the introduction of the perturbation has the clear effect of flattening the spectrum, with the effect being again stronger with increasing amplitude of perturbation. Furthermore, the spectrum at the CP becomes an almost perfect power law, so we can clearly tell when the critical state is reached. Again, the EW becomes clearly enhanced by introducing the perturbation.

### 4.2 Early warnings for the atrial model

#### 4.2.1 Statistical based early warnings

**Fig 6:**
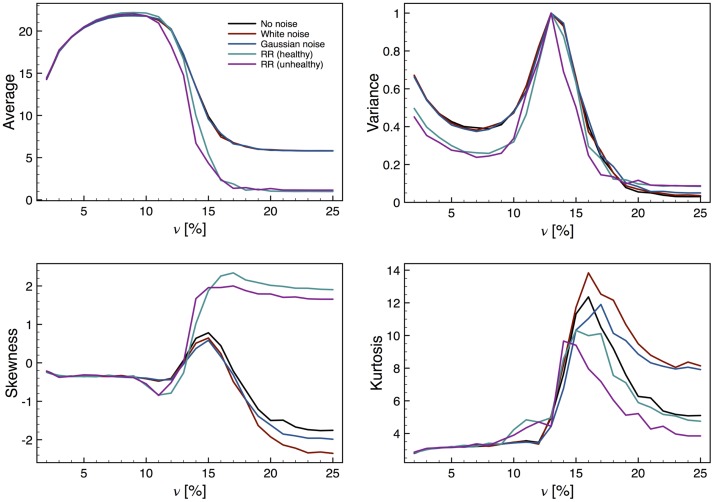
Statistical moments for the atrial model. (a) Average excitation intensity as a function of the percentage of vertical connections, *ν*. A a transition around *ν* = 0.14 is clearly seen, being stronger for more complex perturbations (those of real RR intervals); this means the EW is enhanced. (b) The variance has a peak around the CP, and for more complex perturbations this moment is enhanced as an EW for values of *ν* before the CP. (c) The skewness is a very good EW even without a perturbation, but the fact that when complex perturbations are introduced this parameter changes sign makes it even better. (d) The kurtosis is also a good EW and with a perturbation it can be see that the curve is softened around the CP.

Using the time series generated from the model of atrial tissue, we also studied the statistical moments for an ensemble of simulations for a range of values of *ν*, the percentage of vertical connections. The mean excitation intensity as a function of *ν* is shown in Fig 6, exhibiting an abrupt change around the critical value *ν* = 0.14, in close agreement with Christensen [13]. The colored curves show the effect of introducing a perturbation to the system following the strategies previously described. It can be seen that the perturbation seems to have no effect, as compared with the case with no noise, for the artificial (white and Gaussian) noise strategies. However, introducing a noise of a complex nature, that of real RR intervals, does have a noticeable effect for the behavior past the CP, improving the strength of the EWs. This would mean that an actual, complex biological system may have a greater chance of showing clear EWs compared to simpler theoretical models. Moreover, it suggests that if stimulating a greater RR variability in a patient could improve the chance of detecting the EWs that indicate closeness to a state where atrial fibrillation is more likely to occur.

In Fig 6b we see that the variance has a sharp increase near the critical value of *ν*, indicating that a phase transition occurs around this value. This statistical moment is a good EW because of the smooth change of the curve before and after the transition, which is in contrast with the case of the Kuramoto model. It is observed that the variance increases smoothly on each side of the CP, and again a noticeable difference is present when the noise is taken from real RR intervals.

For the skewness (Fig 6c), for *ν* < 0.14 all cases have a negative value close to zero, and a transition is seen near the CP. But the behavior past the CP is different depending on the presence and type of noise: for no perturbation, or for simple noises, the skewness increases momentarily and then converges towards a negative value, while for the complex noise obtained from RR intervals the skewness converges to a positive value. Regardless of the case, however, the skewness is the best EW we have found for this model since it clearly shows which side of the transition the system is in. Finally, the kurtosis (Fig 6d) is also a good EW, being almost constant for the cases *ν* < 0.14 and exhibiting a sharp change for values above the critical one, regardless of the type of noise used.

#### 4.2.2 Memory based early warnings

**Fig 7:**
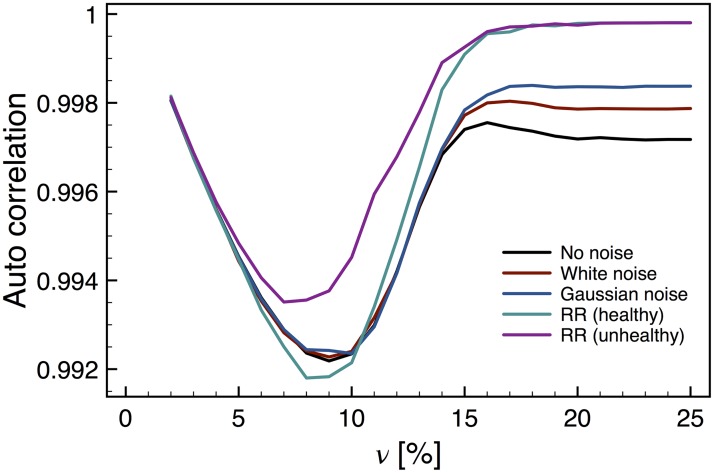
Lag-1 auto correlation for the atrial model. This parameter almost reaches unity at the critical point, albeit it remains nearly constant after the CP. For values of *ν* before the CP the lag-1 correlation is slightly smaller, but the effect may be too small to be significative.

Our results for the short-term memory behavior are shown in Fig 7. The value of the lag-1 autocorrelation is slightly smaller for *ν* < 0.14 than for *ν* > 0.14, which can be interpreted as a loss of short-term memory for low values of *ν*. For *ν* very near the CP the system has the expected behavior, presenting a lag-1 autocorrelation close to unity, which is somehow maintained for values above the critical one. Regrettably, the scale of this change is too small to be significative. It is still worth mentioning that the RR cases for *ν* > 0.14 seem to be closer to 1, which along with the result of the variance indicates that the system goes through a critical transition.

**Fig 8:**
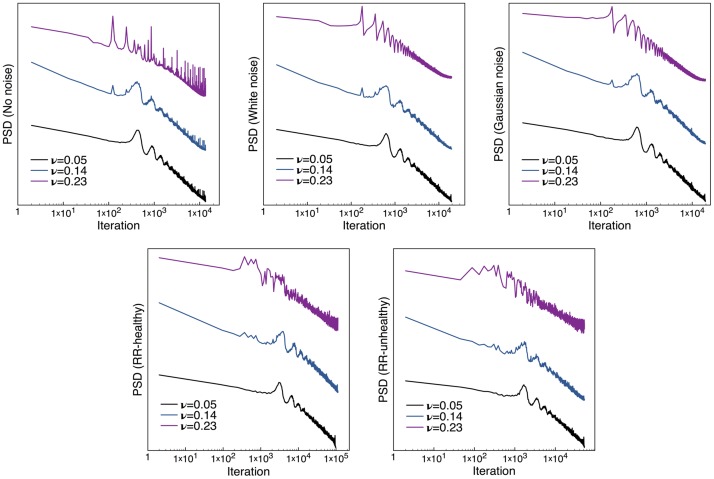
Power spectra for the atrial model. Each panel shows the spectra for different types of perturbations, with curves values of *ν* before, at and after the critical point being compared. The effects of the perturbations are not evident in this case. It could be that, if they exist, they are hidden by the oscillatory nature of the model.

The computed PSD for all cases under study can be seen in Fig 8. For all the types of noise, when *ν* = 0.14 the spectra are slightly closer to single a power law while for other values of *ν* the spectra are better approximated as two distinct power law. The oscillations shown in all spectra are a consequence of the periodic nature with which the excitation pulses are imposed. It is interesting to note that the oscillations seem to be less clear in the PSDs that correspond to noise obtained from actual RR intervals, specially for the *ν* = 0.23. The important fact is the evolution of the spectra that suggests that the system becomes critical near *ν* = 0.14. It is remarkable that introducing a perturbation does not seem to enhance this EW. This could be because the effects of the periodicity with which the pulse is introduced is strong enough to almost hide any perturbation effects.

This model of atrial tissue shows the importance of re-entrant circuits in generating and sustaining atrial fibrillation. As was stated by [13], the findings of the model suggest several ways to prevent the state conducive to fibrillation. One would be to increase the length of the refractory time (through medication), thus stopping the cyclic effect of re-entrant circuits that are below a given length. Another option would be to perform localized ablation of the atrial tissue in regions where re-entrant circuits are suspected, thus preventing their activation altogether.

Our model suggests that early detection of high risk of atrial fibrillation through the study of EWs could be accomplished by measurement and analysis of atrial electric activity. One conceivable way would be through electrocardiographic recording using the lead II configuration, which is known to mainly measure atrial activity, or through the extraction of P-waves from a typical ECG. The resulting timeseries would be analyzed following the methods described here in order to extract the EWs. Then, the proximity (and thus risk) of the transition to the regime of atrial fibrillation of an apparently healthy subject could be assessed by comparing the values of the EWs of multiple cardiac recordings of the same subject obtained over time (when close to the regime transition the EWs diverge or change rapidly, as shown). A long-term better alternative would be to conduct proper clinical characterization of the typical electrical atrial activity (and resulting EWs) by studying groups of healthy control subjects and of subjects presenting AF, which could be the topic of a future study. If such study found clear differences in the EW values between the groups then the healthy range of values for the EWs could be constructed, thus allowing early diagnosis of the risk of AF of a particular subject with a single ECG recording. Some studies are going in this direction and have shown interesting results [17].

## 5 Conclusions

We have performed several studies of EWs for two different systems, an ensemble of oscillators governed by the Kuramoto model and a recent model for the atrial tissue capable of explaining fibrillation. As shown by several previous works [2–6], regardless of its nature a system having a bifurcation in its dynamics can present EWs of a nearby transition. As has been seen here (as well as in the literature), these warnings are not always evident in a single type of analysis, and it is thus generally necessary to probe for both statistics-based and memory-related early warnings [8]. In the case of the models under study here, it is clear that the most useful EWs are the third and the fourth statistical moments and the lag-1 autocorrelation, as seen from smooth behavior of these parameters before and after the critical point and in the non-abrupt changes as the critical point is reached.

In particular, we studied the effect of introducing an external perturbation, in the form of noise of various types and intensities, on the detectable EWs. For the specific case of the Kuramoto model we found that as the intensity of perturbation is increased some EWs are enhanced (with the effects being more readily evident in the memory-based EWs). For the case of the atrial fibrillation model we observed that some of the EWs are improved when complex perturbations are introduced to the system (we found that the skewness and the kurtosis are the parameters most affected). This brings us to the general conclusion that the stronger and more complex the perturbation introduced into the system, the greater effect it has on the EWs, and therefore on our ability to detect them before the critical point is reached. This could offer a great opportunity for the early forecast of an approaching transition in the dynamics of the system.

Our study of the atrial model also showed that the intrinsic complexity of biological signals actually enhance EW properties of these systems. In our case the complexity was obtained by using real RR intervals from the Fantasia database as a source of variability. Finally, the model suggests strategies for improved diagnosis of the risk of atrial fibrillation even before the onset of fibrillation events.

## Supporting information

S1 Fig Atrial model movie. A short video in which we shown how the atrial model works. It is a 50 × 50 grid, in which in the inicial frame the original pulse is shown in red; the blue lines are the vertical connected cells and the cell in black is the one which fails conductivity. It can be seen how, when the black cell blocks the pulse and due to the topology of the net, the re-entry pulse is generated giving place to the elliptical wave fronts.
(MOV)
